# Prolonging Shelf Life and Meat Quality of Rainbow Trout ( *Oncorhynchus mykiss* ) by Immersing in Pine Nut ( *Pinus gerardiana* ) Extract During Cold Storage

**DOI:** 10.1002/fsn3.4685

**Published:** 2024-12-19

**Authors:** Shaghayegh Soleimani, Shabnam Haghighat Khajavi, Reza Safari

**Affiliations:** ^1^ Department of Food Science and Technology, Science and Research Branch Islamic Azad University Tehran Iran; ^2^ Caspian Sea Ecology Research Center Iranian Fisheries Science Research Institute, Agricultural Research, Education and Extension Organization Mazandaran Iran

**Keywords:** antioxidant activity, meat quality enhancement, microbial spoilage, pine nut ( *Pinus gerardiana* Wallich), rainbow trout ( *Oncorhynchus mykiss* ), shelf‐life extension

## Abstract

Rainbow trout ( *Oncorhynchus mykiss* ) is a freshwater fish susceptible to chemical and microbial spoilage, limiting its shelf life. This study aimed to enhance and extend the rainbow trout fillets' shelf life stored at 4°C ± 1°C through an immersion treatment using ultrasound‐assisted, defatted pine nut (*Pinus gerardiana* Wallich) extracts at concentrations of 1% and 2% (w/v), compared to the control group (0% pine nut). Evaluations were conducted at storage intervals of 0, 4, 8, 12, 16, and 20 days. The methodology assessed antioxidant activity through 2,2‐diphenyl 1‐picrylhydrazyl radical scavenging, which showed a linear increase with pine nut extract concentration, reaching 59.24% at 2%. Chemical indicators, such as peroxide values, thiobarbituric acid values, free fatty acids, and total volatile basic nitrogen, decreased significantly (*p* ≤ 0.05) with higher concentrations of pine nut extract, with the lowest values recorded at 2% across all storage days. Microbial analysis showed a significant reduction (*p* ≤ 0.05) in the total viable count, psychrotrophic bacteria count, lactic acid bacteria, Enterobacteriaceae, and H₂S‐producing bacteria with increasing pine nut concentrations, with the 2% treatment yielding the lowest microbial loads throughout storage. Sensory evaluation indicated that higher pine nut concentrations improved the acceptability of color, odor, and taste (*p* ≤ 0.05). However, significant degradation (*p* ≤ 0.05) in chemical, microbial, and sensory parameters occurred with prolonged storage duration. In conclusion, the 2% pine nut extract was the most effective immersion treatment for extending the shelf life of rainbow trout fillets for up to 12 days.

## Graphical abstract

This study assessed the impact of 1% and 2% pine nut extract on the shelf life of rainbow trout during cold storage (4°C ± 1°C). The 2% extract significantly improved antioxidant activity, reduced peroxide, thiobarbituric acid, free fatty acid, and total volatile basic nitrogen values, and lowered microbial loads. Sensory acceptance for color, odor, and taste also increased with higher extract concentrations. The 2% pine nut extract was most effective, extending the shelf life of rainbow trout fillets to 12 days.

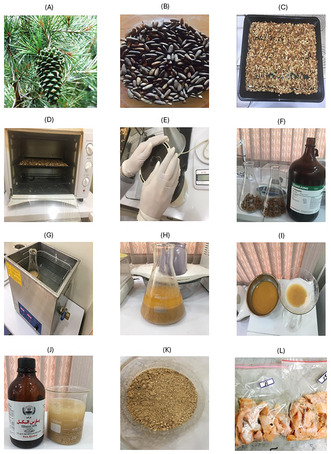

## 1 Introduction

Maintaining the freshness and meat quality of rainbow trout ( *Oncorhynchus mykiss* ), which are a member of the Salmonidae family and commonly farmed species in Iran due to their rapid growth and adaptability to various temperatures and water qualities (Oraei et al. 2011) during storage is a significant issue for the food industry due to their susceptibility to chemical and microbial spoilage. The fatty acid composition of rainbow trout varies significantly depending on its diet and typically contains high levels of monounsaturated (29.6%–45.2%) and polyunsaturated fatty acids (31.9%–36.4%; Trbović et al. 2012). Edible coatings and films made from proteins, polysaccharides, or lipids block moisture, oxygen, and solutes, enhancing food preservation (Senturk Parreidt, Müller, and Schmid 2018; Zhang et al. 2020). Incorporating antioxidant and antimicrobial agents improves their effectiveness, particularly for farmed fish (Zhou et al. 2024). For instance, gelatin films infused with propolis extract extended the sensory acceptability of rainbow trout fillets to 15 days under refrigeration, compared to just 9 days for uncoated fillets (Uçak et al. 2020). Additionally, coatings that combine the lactoperoxidase system with chitosan have been shown to delay the development of fishy odors by reducing microbial activity (Jasour et al. 2015). The spoilage primarily results from the high polyunsaturated fatty acid content, which is prone to lipid oxidation. Lipid oxidation and microbial growth deteriorate sensory attributes, including flavor, odor, and texture, causing nutritional losses and ultimately affecting consumer acceptance while increasing food waste (Nie et al. 2022; Wang et al. 2023). Thus, extending the rainbow trout fillet's shelf life is crucial for maintaining quality and meeting consumer demands.

Various preservation strategies, such as edible films, coatings, and the use of antioxidant compounds, have been studied to prolong the shelf life of fish products, especially those rich in polyunsaturated fatty acids (Pirozzi et al. 2021; Suhag et al. 2020; Nobahar, Haghighat Khajavi, and Safari 2022; Uçak, Özogul, and Durmuş 2011; Volpe et al. 2015). Plant‐based natural antioxidants, including essential oils and herbal extracts, have demonstrated effectiveness in reducing lipid oxidation and microbial spoilage in fish fillets, thereby enhancing shelf life (Shahidi and Ambigaipalan 2015; Takyar, Haghighat Khajavi, and Safari 2019; Amorati, Foti, and Valgimigli 2013; Oroian and Escriche 2015; Tajkarimi and Ibrahim 2012). However, despite their potential, pine nut extracts, particularly from *Pinus gerardiana* , have been limitedly investigated in this regard, even though they are known for their antioxidant and antimicrobial properties (Hoon et al. 2015; Shahidi and Alsalvar 2008).

There is no universally accepted definition of nuts in food commodities, and CN codes identify fruits classified as nuts. For example, as per Commission Implementing Regulation (EU) 2017, no.1925, pine nuts (code 0802 90 50) are defined as “decorticated kernels of various species of Gymnosperm, Pinus: e.g., *pinea*, *koraiensis*, *sibirica*, *yunnanensis*, *wallichiana*, *gerardiana*, and *pumila*.” Pine nuts are prevalent and widely consumed in various cultures, particularly in the Mediterranean, Middle Eastern, Asian, and southwestern United States regions (Yu and Slavin 2008). Among these, *Pinus gerardiana* Wallich ex. D. Don, also known as Chilgoza pine, is recognized as a medicinal plant with high polyphenolic content that provides antioxidant benefits (Hoon et al. 2015; Shahidi and Alsalvar 2008). This species, found in regions like Pakistan, Northern Kashmir, Eastern Afghanistan, and Northwest India, has a lipid content of 52.1%, primarily comprising monounsaturated and polyunsaturated fatty acids, especially linoleic and oleic acids. Additionally, it contains 13.6% protein, 13.1% carbohydrate, 4.1% sugar, 2.0% moisture, and 3.1% minerals and ash (Sathe et al. 2008).

Nuts are abundant in various phenolic compounds that hold significant biological functions. These compounds can be classified into five main categories: phenolic acids, flavonoids, tannins, phenolic lignans, and stilbene derivatives. Due to their complex composition, it is challenging to establish a single extraction method that is effective for all nut phenolics. Innovative extraction techniques, such as microwave, ultrasound, and compressed fluid solvents generally recognized as safe, are gaining popularity (Bodoira and Maestri 2020).

The bioactive compounds in *Pinus gerardiana* , such as gallocatechin, catechin, gallic acid, lycopene, carotenoid, and phytosterols, provide multiple health benefits, including antioxidant, anticancer, anti‐inflammatory, antiviral, and cholesterol‐lowering effects (Singh, Kumar, and Dash 2021). This makes pine nuts a promising choice for natural food preservation, particularly for rainbow trout fillets.

Given the limited exploration of pine nut defatted extracts as water‐soluble antioxidants (flavonoids and phenolic acids) in fish preservation, this study aims to evaluate the effectiveness of ultrasound‐assisted immersion treatment using defatted pine nut extracts at concentrations of 1% and 2% (w/v) to extend rainbow trout's fillet shelf life, stored at 4°C ± 1°C. By comparing this approach with other methods documented in the literature, the research provides a novel perspective on using *Pinus gerardiana* in food preservation.

## 2 Materials and Methods

### 2.1 Chemicals and Reagents

Ethanol (Pars alcohol, Iran), n‐hexane, 1‐butanol, chloroform, 1,1‐diphenyl‐2‐picrylhydrazyl (DPPH), gallic acid, acetic acid, sodium thiosulfate, potassium hydroxide, methyl red, bromocresol green, thiobarbituric acid, boric acid, hydrochloric acid, potassium iodide, starch, phenolphthalein, and magnesium oxide were all procured from Merck in Germany. In addition, Plate Count Agar (PCA), Violet Red Bile Agar (VRBA), Iron Agar, and CFC Selective Agar were purchased from Merck.

### 2.2 Samples Collection

The pine nuts were imported from Afghanistan and purchased from Iran's local market in September. Rainbow trout were obtained from an aquaculture farm in Iran.

### 2.3 Sample Preparation

#### 2.3.1 Pine Nut Oil Extraction

The pine nut seeds were de‐shelled, with the kernel and brown skin separated. Afterward, the samples were dried, crushed, and ground into a powder using a steel grinder for 30 s, resulting in a particle size of less than 0.5 mm. The powdered pine nuts were mixed with n‐hexane in a conical flask using a 1:5 (w/v) ratio. The extraction process was facilitated using ultrasound (Langford Sonomatic, UK) for 30 min at 40°C with continuous stirring to separate the oil from the pine nut seeds, resulting in the defatted pine nut extract. The resulting mixture was left in a capped separatory funnel overnight. After 24 h, the sediment was decanted from the pine nut oil. The defatted pine nut was then filtered using Whatman No.1 filter paper, and the solvent was subsequently evaporated at 40°C using a rotary vacuum evaporator (EYELA Auto jack NAJ‐100, Japan). Finally, the extract (defatted nut) was placed in a desiccating chamber at an ambient temperature of 24 h to remove any residual n‐hexane (Bligh and Dyer 1959).

#### 2.3.2 Pine Nut Extract Preparation

Ultrasound‐assisted extraction was conducted on defatted pine nut seed powder. The powder was combined with ethanol in a 1:10 (w/v) ratio in a conical flask, continuously stirred, and sonicated in an ultrasonic bath (Langford Sonomatic, UK) at a frequency of 40 kHz for 20 min at room temperature to ensure effective solvent/extract interaction. The subsequent step involved filtering the extract using Whatman No.1 filter paper and then using a rotary evaporator to concentrate it at 40°C under vacuum. The resulting pine nut kernel extract, devoid of fat, was then dried into a powder using oven hot‐air flow at 40°C (Thilakarathna et al. 2023).

#### 2.3.3 Fish Fillets Preparation and Treatment With Pine Nut Extract

**FIGURE 1:**
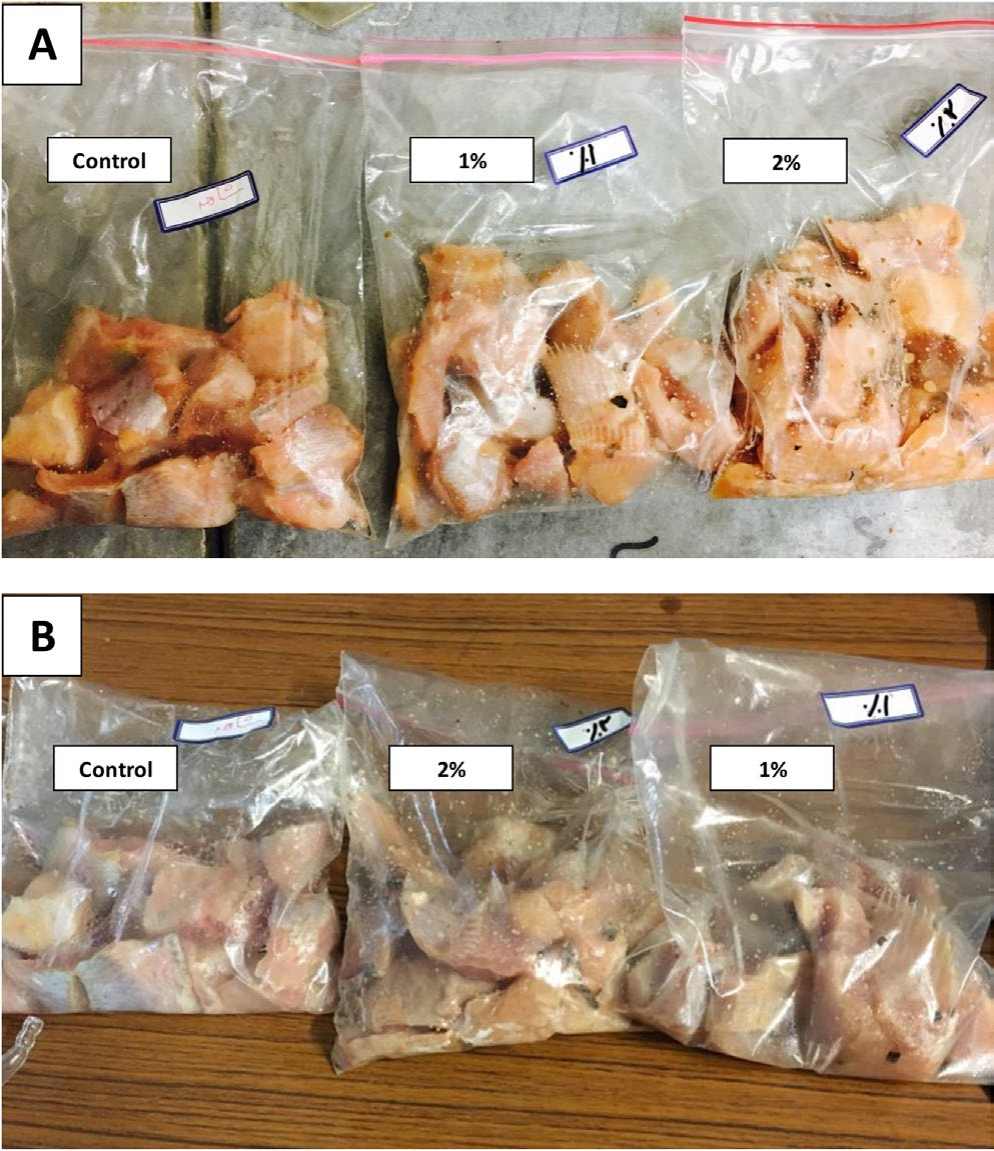
Control and immerse treated rainbow trout fillets with Pinus gerardiana extract. (A) Control and treated samples at day 0 of storage. (B) Control and treated samples at day 12 of storage.

Rainbow trout, about 350–400 g on average, were obtained from fish farms, cooled on ice, and then transferred to the laboratory. The fish were then carefully prepared by removing the head, bones, and skin and then hand‐filleted. Following a thorough wash with tap water, the fillets were precisely cut into 5 × 5 cm squares with a 3 cm thickness to ensure uniformity. The fish fillet samples were divided into three batches: one control sample (uncoated) and two treatment samples with concentrations of 1% and 2% of ultrasound‐assisted defatted extract of pine nuts (Figure 1A). The fish fillets were immersed in the defatted pine nut extract at 1% and 2% concentrations for 2 min and then allowed to drain to prepare the coated samples. All treatments were prepared in triplicate and packaged in sterile, hand‐sealed zip‐lock polyethylene bags to create a semi‐vacuum condition. The packages were labeled and stored in the refrigerator at 4°C ± 1°C for 20 days. Chemical, microbial, and sensory analyses were conducted at 4‐day intervals during storage to assess the fish quality.

### 2.4 Chemical Analysis

#### 2.4.1 Antioxidant Activity Determination by 1,1‐Diphenyl‐2‐Picrylhydrazyl (DPPH‐RSA) Radical Scavenging Activity Assay

The DPPH radical scavenging activity of pine nut extract was assessed following the method outlined by Brand‐Williams, Cuvelier, and Berset (1995) with minor adjustments. A stock solution of 0.1 mM DPPH radical solution in 96% ethanol was prepared with gallic acid as a standard. Various concentrations of defatted pine nut kernel extracts (0.25%, 0.5%, 1%, and 2% w/v) were prepared in ethanol. The mixture was centrifuged at 5000 rpm for 20 min, and the supernatant was then gathered. Subsequently, 1.5 mL of each ethanolic pine nut extract sample solution was mixed with an equal amount of ethanolic DPPH solution, vortexed for 1 min, and left at room temperature in darkness for 30 min. Absorbance was measured at 517 nm using a UV/Vis spectrophotometer (UNICO UV‐2100, USA), with a mixture of 0.1 mM DPPH radical solution and 1.5 mL of 96% ethanol as the blank. The inhibition percentage of DPPH by the samples was calculated using the following equation: Ab and As represent the blank and sample absorptions, respectively.

$$\text{DPPH Radical Scavenging Activity}\%=\frac{A_{\text{blank}}-A_{\text{Sample}}}{A_{\text{blank}}}\times 100$$

#### 2.4.2 Peroxide Value (PV) Determination

The peroxide value (PV) was examined using the AOCS official method (AOCS 2017a), which involves measuring the meq of H_2_O_2_ in kilograms of fat. The equation uses the volume (V) of sodium thiosulfate (mL), the normality (N) of the solution, and the weight (W) of the fish oil (g).

$$\mathrm{PV}=\frac{V\times N\times 1000}{W}$$

#### 2.4.3 Thiobarbituric Acid Reactive Substances (TBARS) Determination

The colorimetric method assessed the TBA values per the method outlined by the AOCS official method (AOCS 2017b). In a 25 mL volumetric flask, 1‐butanol was added to 200 mg of minced fish meat and then brought to volume and vortexed. After filtration, 5 mL of this mixture was added to a test tube, mixed with 5 mL of TBA reagent, capped, and incubated at 95°C for 2 h in a water bath. Subsequently, the mixture was brought to ambient temperature, and the sample's absorbance was measured at 530 nm using a UV/Vis spectrophotometer. Distilled water served as the blank sample for this measurement. The TBA value was then calculated using the equation as mg of malondialdehyde (MDA) per kg of fish meat. Abs_Sample_ and Abs_Blank_ represent the sample's absorption and the blank, respectively.

$$\text{TBARS}=\frac{\left({\mathrm{Abs}}_{\text{sample}}-{\mathrm{Abs}}_{\text{blank}}\right)}{200}\times 50$$

#### 2.4.4 Total Volatile Basic‐Nitrogen (TVB‐N) Determination

The TVB‐N value of the fish sample was determined according to European Union Commission Regulation (EC) No 2027/2005. The sample, consisting of 10 g of minced fish, was homogenized and then subjected to distillation after the addition of 2 g of magnesium oxide. Steam distillation was done using a Kjeldahl Apparatus. The digested distilled sample was collected in ethanol in a flask that contained 10 mL of 2% boric acid and a mixed indicator solution of bromocresol green/methyl red (1:1 v/v). An aqueous acid solution absorbed the volatile basic components, leading to a green color change. The boric acid was titrated with 0.1 mol/L hydrochloric acid until completely neutralized. A color change to pink indicated the endpoint by adding a single drop of hydrochloric acid. The TVB‐N value was then expressed as mg of nitrogen per 100 g fish sample.

#### 2.4.5 Free Fatty Acids (FFA) Determination

The fish oil's free fatty acid content was determined using the acidimetric titration method and exhibited as a percentage of oleic acid. The calculations were performed in accordance with the AOCS official method (AOCS 2017c), utilizing the equation where V represents the volume (mL) of the KOH solution, N is the solution normality, and W is the fish oil sample weight (g).

$$\mathrm{FFA}=\frac{V\times N\times 28.2}{W}$$

### 2.5 Microbiological Analysis

10 g of each control and treated sample with 1% and 2% pine nut extract were aseptically measured and placed into separate sterile sampling bags. After that, they were diluted with 90 mL of 0.1% (w/v) peptone water. The samples were homogenized using a stomacher (Stomacher 400 Circulator, UK) at 230 rpm for 2 min. Afterward, appropriate serial dilutions were made from this homogenized sample, and 0.1 mL from each prepared dilution was spread onto the specified microbial culture media.

Microbiological analysis was conducted to assess the quality of the fish fillets. The pour plate method with Plate Count Agar (PCA) was utilized to determine the total viable count (TVC), and the samples were incubated at 30°C for 72 h (ISO 4833‐1, 2022). Psychrotrophic bacteria count (PBC) was determined using PCA at 7°C for 10 days (Song, Shin, and Song 2012). Lactic acid bacteria (LAB) count was performed using De Man‐Rogosa‐Sharpe Agar (MRS) after incubating at 30°C for 72 h. Enterobacteriaceae count was determined using Violet Red Bile Agar (VRBA) after incubating at 36°C for 48 h (ISO 21528‐2 2004). H_2_S‐producing bacteria were determined using Iron Agar after incubating at 25°C for 5 days using the pour plate method. Pseudomonas count was determined using Pseudomonas Selective Agar (CFC) after incubating at 25°C for 48 h. All microbial counts were performed in triplicate, and the microbial fish fillet results were expressed as log_10_ CFU/g.

### 2.6 Sensory Evaluation

Samples for the organoleptic test were steam‐cooked at 60°C for 30 min, and the sensory characteristics of cooked control and treated fish samples were evaluated during the entire storage period based on a five‐point hedonic scale rating (1 = dislike extremely to 5 = like extremely; Lim 2011). Ten trained panelists aged between 25 and 50 scored the quality parameters, including the samples' color, odor, and taste.

### 2.7 Statistical Analysis

The statistical analysis was conducted in triplicate for each treatment at specific storage times. The data was analyzed using SPSS version 22.0 software (IBM, USA) for one‐way analysis of variance (ANOVA). Duncan's multiple‐range tests were used for means comparison to assess significant differences (*p* ≤ 0.05). The sensory score was also analyzed using the non‐parametric Kruskal–Wallis and Mann–Whitney U tests to identify any differences. All statistical analyses were performed at a significance level of *p* ≤ 0.05, and the results were reported as mean ± standard deviation.

## 3 Results and Discussion

### 3.1 Antioxidant Activity (DPPH‐RSA)

**FIGURE 2:**
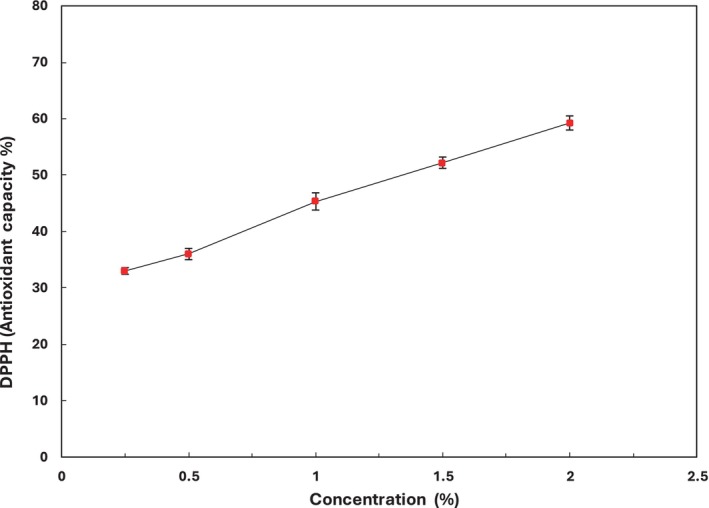
Scavenging effects of *Pinus gerardiana* on DPPH radical.

The antioxidant activity of the defatted pine nut ethanolic extract was evaluated using the DPPH assay (Table 1). The DPPH radical scavenging activity increased significantly (*p* ≤ 0.05) with the concentration of the extract (Figure 2), reaching a 59.24% inhibition at a concentration of 2 mg/mL. Hoon et al. (2015) noted the presence of various phenolic compounds in pine nut extract, including gallocatechin, catechin, lutein, gallic acid, lycopene, carotenoids, and tocopherols. Consequently, the high antioxidant activity of pine nut extract may be attributed to these phenolic compounds, which act as radical scavengers, potentially inhibiting lipid oxidation in refrigerated fish. The highest level of DPPH free radical scavenging activity was detected at a 2% concentration of pine nut extract, indicating a linear relation between the quantity of pine nut extract and its antioxidant activity. The antioxidant activity of a compound is commonly expressed as IC50, which represents the concentration required to inhibit oxidation or reduce the initial DPPH concentration by 50%. This serves as a measure of the compound's efficacy as an antioxidant. The results indicated that the IC50 value was 136.72 μg/mL. However, a study by Sharma, Goyal, and Sharma (2016) reported an IC50 value of 102.86 μg/mL. The higher IC50 value observed in our study may be due to the beneficial effects of ultrasonic‐assisted extraction on the antioxidant properties of the aqueous extract.

**TABLE 1:** Pine nut extract radical scavenging activity.

Concentrations (% w/v)	DPPH activity %
0.25	33.00 ± 0.58^a^
0.5	36.00 ± 1.00^b^
1	45.33 ± 1.53^c^
1.5	52.20 ± 1.00^d^
2	59.24 ± 1.25^e^

*Note:* Data are represented as Mean ± Standard deviation (*n* = 3). Different letters indicate the significant differences between the treatment groups (*p* ≤ 0.05).

### 3.2 Peroxide Value (PV)

**FIGURE 3:**
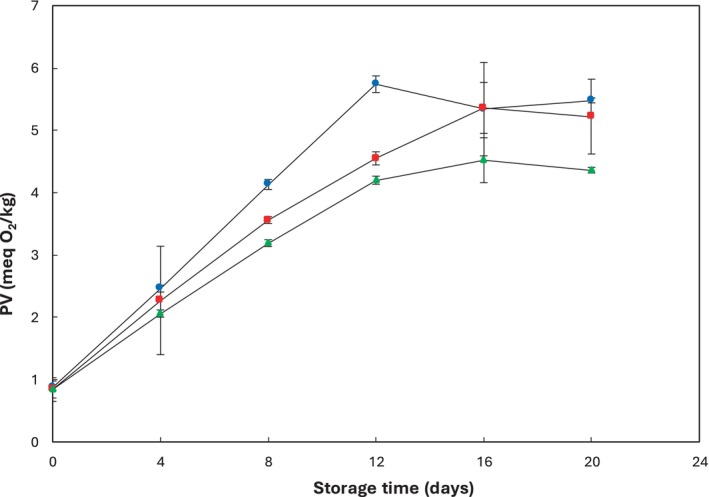
Changes in peroxide value (PV) of rainbow trout fillets stored at 4°C ± 1°C for 20 days. Values are given as mean ± standard deviation (*n* = 3). Control, fillets without *P. gerardiana* (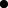); PG 1%, fillets contained 1% *P. gerardiana* (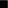); PG 2% fillets contained 2% *P. gerardiana* (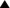).

The study examined the variations in peroxide value to assess the storage conditions and shelf life of rainbow trout, a fish rich in polyunsaturated fatty acids and prone to oxidation (Kołakowska et al. 2006). This measure detects hydroperoxides, which are primary products of lipid oxidation (Rusted 2009; Sallam et al. 2007). The initial peroxide values of the fish fillet samples were found to be 0.88 meq/kg, consistent with the findings of Pezeshk, Rezaei, and Hosseini (2011) and Ojagh et al. (2010). The results indicated significant differences (*p* ≤ 0.05) between the control and samples containing 1% and 2% concentrations of pine nut extract on day 4 (Figure 3). The PV of samples initially increased, and this increase was significant (*p* ≤ 0.05) for the control sample (5.74 meq/kg) compared to samples containing 1% (4.55 meq/kg) and 2% (4.20 meq/kg) of pine nut extract on day 12 and then gradually decreased (Table 2). This pattern of increase and subsequent decrease aligns with the findings of Boselli et al. (2005), who noted the breakdown of lipid hydroperoxides into secondary oxidation products like aldehydes, ketones, and other volatile compounds, contributing to a decrease in flavor and odor in fish (Goulas and Kontominas 2007a; Uçak, Özogul, and Durmuş 2011). The peroxide values of the samples showed a slight increase on day 16 and remained relatively constant from day 16 to 20 in both the control and treated samples. However, fish fillet samples containing 2% pine nut extract exhibited lower peroxide values compared to samples with 1% extract and the control throughout the storage period (*p* ≤ 0.05). These findings demonstrated that phenolic antioxidants from the higher concentration of pine nut extract effectively absorbed oxygen (Mohan, Ravishankar, and Srinivasagopal 2008), inhibited the formation of free radicals, and delayed the autoxidative process in rainbow trout fillets during refrigerated storage (O'Sullivan et al. 2005). Aligning with this study, Mi, Guo, and Li (2016) reported that 6‐gingerol, particularly at higher concentrations, significantly inhibited lipid oxidation in red drum fillets during cold storage.

**TABLE 2:** Chemical analysis of rainbow trout fillets including control sample and samples contained pine nut extract during 20 days of storage at 4°C ± 1°C.

Analysis	Treatments	Storage days					
		0	4	8	12	16	20
PV (meq O^2^/kg)	Control	0.88 ± 0.042^Ae^	2.46 ± 0.055^Ad^	4.13 ± 0.080^Ac^	5.74 ± 0.133^Aa^	5.34 ± 0.075^Ac^	5.48 ± 0.040^Ab^
	1%	0.84 ± 0.136^Af^	2.27 ± 0.087^Be^	3.56 ± 0.055^Bd^	4.55 ± 0.105^Bc^	5.36 ± 0.041^Aa^	5.22 ± 0.060^Bb^
	2%	0.84 ± 0.019^Af^	2.06 ± 0.058^Ce^	3.19 ± 0.055^Cd^	4.20 ± 0.065^Cc^	4.52 ± 0.036^Ba^	4.36 ± 0.045^Cb^
TBA (mg MDA/kg)	Control	0.72 ± 0.025^Af^	1.51 ± 0.041^Ae^	1.88 ± 0.091^Ad^	2.74 ± 0.113^Ac^	3.60 ± 0.162^Ab^	4.14 ± 0.474^Aa^
	1%	0.72 ± 0.029^Af^	1.34 ± 0.030^Be^	1.44 ± 0.043^Bd^	2.33 ± 0.170^Bc^	3.26 ± 0.105^Bb^	3.73 ± 0.105^Ba^
	2%	0.70 ± 0.060^Af^	1.15 ± 0.052^Cd^	0.83 ± 0.496^Cc^	1.87 ± 0.512^Cc^	2.24 ± 0.115^Cb^	2.58 ± 0.110^Ca^
TVB/N (mg/ 100 g)	Control	10.47 ± 0.17^Af^	21.23 ± 1.27^Ae^	30.63 ± 0.48^Ad^	48.04 ± 0.69^Ac^	62.56 ± 0.94^Ab^	72.50 ± 0.778^Aa^
	1%	10.39 ± 0.12^Af^	16.93 ± 0.55^Be^	27.31 ± 0.54^Bd^	42.91 ± 0.68^Bc^	51.34 ± 0.99^Bb^	69.09 ± 1.745^Ba^
	2%	10.22 ± 0.09^Af^	14.87 ± 0.49^Ce^	24.02 ± 0.52^Cd^	29.12 ± 0.31^Cc^	40.32 ± 1.91^Cb^	55.02 ± 1.57^Ca^
FFA (% of Oleic acid)	Control	0.53 ± 0.03^Af^	0.98 ± 0.040^Ae^	2.29 ± 0.103^Ad^	3.42 ± 0.098^Ac^	4.16 ± 0.046^Ab^	4.91 ± 0.098^Aa^
	1%	0.47 ± 0.03^Af^	0.92 ± 0.086^Ae^	2.20 ± 0.103^Ad^	3.33 ± 0.092^Ac^	4.20 ± 0.098^Ab^	4.62 ± 0.075^Ba^
	2%	0.49 ± 0.05^Af^	0.79 ± 0.069^Be^	1.75 ± 0.063^Bd^	2.24 ± 0.092^Bc^	3.24 ± 0.051^Bb^	4.05 ± 0.086^Ca^

*Note:* Data are represented as Mean ± Standard deviation (*n* = 3). Different letters indicate significant differences between the treatment groups (*p* ≤ 0.05), with capital letters representing significant differences in treatments and small letters representing storage conditions.

### 3.3 Thiobarbituric Acid Reactive Substances (TBARS)

**FIGURE 4:**
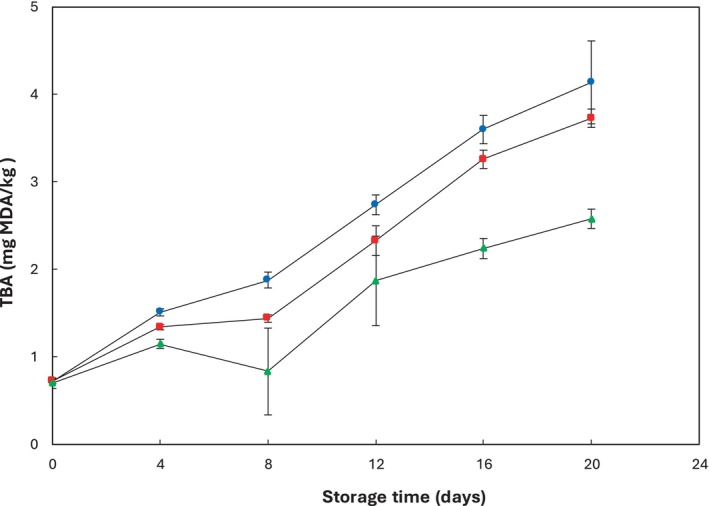
Changes in TBA reactive substances of rainbow trout fillets stored at 4°C ± 1°C for 20 days. Values are given as mean ± standard deviation (*n* = 3). Control, fillets without *P. gerardiana* (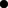); PG 1%, fillets contained 1% *P. gerardiana* (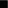); PG 2% fillets contained 2% *P. gerardiana* (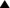).

The TBARS assay is commonly utilized to quantify the malondialdehyde (MDA) content generated from the degradation of lipid hydroperoxides. MDA is a well‐known secondary lipid oxidation product (Goulas and Kontominas 2007b). Elevated TBA values in fish and seafood products lead to reduced consumer acceptance due to the potential interaction of MDA with other fish components, resulting in the formation of secondary metabolites (Ólafsdóttir and Jónsdóttir 2009; Botsoglou et al. 1994) that can negatively impact product quality by causing off‐odors and taste alterations. The results of TBA values for both the control and samples containing 1% and 2% pine nut extract are depicted in Figure 4. As illustrated, a noteworthy increase in TBA values was observed for all samples throughout the entire storage period (*p* ≤ 0.05). Despite the significant increase (*p* ≤ 0.05) in TBA values for control samples and fish fillets treated with 1% pine nut extract, there was a higher increase in the control samples compared to the treated samples. However, samples treated with 2% pine nut extract exhibited significantly lower values. The results indicated that pine nut extract at a concentration of 2% effectively inhibited lipid oxidation by reducing MDA levels in fish fillet samples during refrigerated storage (Table 2). This finding aligns with previous observations by Kornsteiner, Wagner, and Elmadfa (2006) and Miraliakbari and Shahidi (2008), suggesting that phenolic compounds in pine nuts can serve as antioxidants to inhibit oxidation.

### 3.4 Total Volatile Basic Nitrogen (TVB‐N)

**FIGURE 5:**
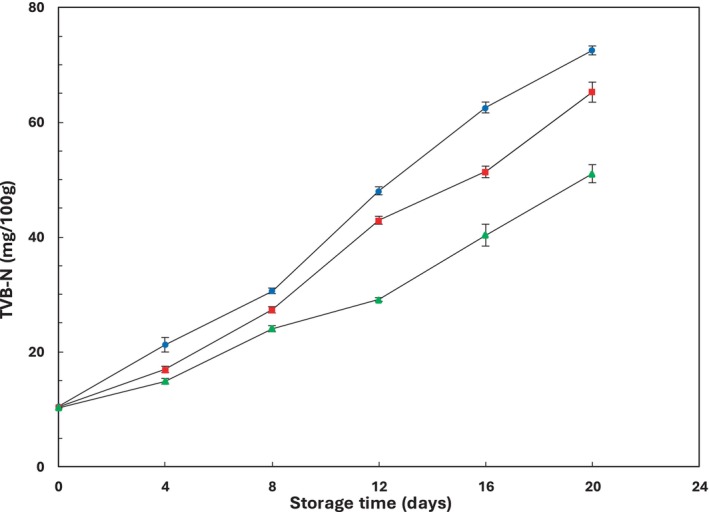
Changes in total volatile basic nitrogen (TVB‐N) of rainbow trout fillets stored at 4°C ± 1°C for 20 days. Values are given as mean ± standard deviation (*n* = 3). Control, fillets without *P. gerardiana* (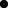); PG 1%, fillets contained 1% *P. gerardiana* (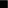); PG 2% fillets contained 2% *P. gerardiana* (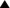).

The TVB‐N change is an important indicator used to determine the quality of fish and seafood products during storage. An increase in TVB‐N value suggests the breakdown of proteins due to the activity of proteolytic bacteria and hydrolysis by endogenous enzymes. This breakdown leads to microbial spoilage and the production of trimethylamine, dimethylamine, ammonia, and other volatile compounds (Ghaly et al. 2010). As per European regulations (EC 2074/2005) and (EC 1022/2008), certain fishery products must have a maximum allowable TVB‐N of 25 to 35 mg N/100 g. While there are no specific limits of acceptability for rainbow trout in these regulations, there is a general agreement, recommended by Giménez, Roncalés, and Beltrán (2002) and Arashisar et al. (2004), that 25 mg N/100 g of TVB‐N in trout flesh is an acceptable limit. However, the reliability of TVB‐N as an indicator of fish freshness has been questioned. Recent evidence suggests that fish may still fall within the acceptable TVB‐N range even when sensory evaluation deems it unsuitable for consumption (Alirezalu et al. 2021; Chytiri et al. 2004; Erkan 2012). As reported by Giménez, Roncalés, and Beltrán (2002), initial TVB‐N values for control and samples treated with 1% and 2% pine nut extracts were 10.47, 10.39, and 10.22 mg N/100 g of fish flesh, respectively. Both control and treated samples showed a significant increase (*p* ≤ 0.05) in TVB‐N values with storage time (Figure 5). However, the results indicated that the TVB‐N values for fish fillet samples containing 2% pine nut extract were significantly (*p* ≤ 0.05) lower compared to the control and samples treated with 1% extract (Table 2). Consistent with Hoon et al. (2015) and Fan, Chi, and Zhang (2008), this lower TVB‐N value is attributed to the polyphenolic compounds' inhibitory effect in high concentrations of pine nut extract, which can counteract bacterial growth and/or reduced oxidative deamination of non‐protein nitrogenous compounds.

### 3.5 Free Fatty Acids (FFA)

**FIGURE 6:**
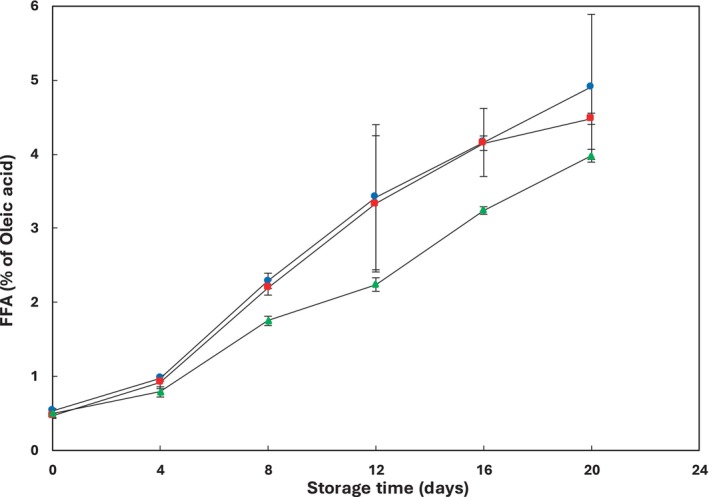
Changes in free fatty acids (FFA) of rainbow trout fillets stored at 4°C ± 1°C for 20 days. Values are given as mean ± standard deviation (*n* = 3). Control, fillets without *P. gerardiana* (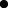); PG 1%, fillets contained 1% *P. gerardiana* (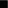); PG 2% fillets contained 2% *P. gerardiana* (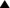).

An increase in FFA contents of fish during cold storage is attributed to enzymatic hydrolysis of lipids, and it has been utilized as an indicator of deterioration (Barthet, Gordon, and Daun 2008; Quitral et al. 2009). Depending on the source of lipids, FFAs can either accelerate or inhibit lipid oxidation (Wu et al. 2021; Secci and Parisi 2016; Pearson et al. 1983; Shewfelt 1981). There is a correlation between lipolysis and lipid oxidation since FFAs are more prone to oxidation compared to esterified fatty acids (Nilsson and Gram 2002). Elevated levels of FFAs lead to diminished freshness, reduced organoleptic acceptability, and a shorter shelf life of fish (Barthet, Gordon, and Daun 2008). The results obtained from the chemical analysis (Table 2) indicated that the level of FFA in both the control and treated samples exhibited significant increases at different rates during refrigerated storage (*p* ≤ 0.05). The findings revealed that the FFA levels in samples containing 2% pine nut extract were lower than those containing 1% and the control samples throughout the storage period (Figure 6). This aligns with the findings of Hoon et al. (2015), who reported that pine nut extract effectively inhibited lipid oxidation and hydrolysis in refrigerated rainbow trout fillets.

### 3.6 Microbiological Analysis

The changes in microbial counts of rainbow trout fillets, including Total Viable Count (TVC), Psychrotrophic Bacteria Count (PBC), Lactic Acid Bacteria (LAB), Enterobacteriaceae, and Hydrogen Sulfide (H_2_S)‐Producing Bacteria, were measured at 4‐day intervals (0 to 20 days) of storage at 4°C ± 1°C (Table 3).

**TABLE 3:** Microbiological analysis of rainbow trout fillets including control sample and samples containing pine nut extract during 20 days of storage at 4°C ± 1°C.

Microorganisms	Treatments	Storage days					
		0	4	8	12	16	20
Total viable count (log10 CFU/g)	Control	3.25 ± 0.050^Af^	5.18 ± 0.075^Ae^	7.12 ± 0.020^Ad^	8.61 ± 0.070^Ad^	9.33 ± 0.072^Ab^	10.36 ± 0.045^Aa^
	1%	3.19 ± 0.055^Af^	5.06 ± 0.185^Be^	6.30 ± 0.020^Bd^	7.36 ± 0.045^Bc^	7.88 ± 0.216^Bb^	8.53 ± 0.104^Ba^
	2%	3.17 ± 0.075^Af^	4.43 ± 0.096^Ce^	5.71 ± 0.060^Cd^	6.52 ± 0.125^Cc^	7.23 ± 0.081^Cb^	7.43 ± 0.088^Ca^
Psychrotrophic bacteria count (log10 CFU/g)	Control	3.31 ± 0.036^Af^	5.34 ± 0.081^Ae^	7.23 ± 0.062^Ad^	8.78 ± 0.062^Ac^	9.52 ± 0.080^Ab^	10.54 ± 0.060^Aa^
	1%	3.28 ± 0.070^Af^	4.89 ± 0.073^Be^	5.45 ± 0.130^Bd^	6.21 ± 0.045^Bc^	6.86 ± 0.110^Bb^	7.54 ± 0.090^Ba^
	2%	3.29 ± 0.056^Af^	4.26 ± 0.055^Ce^	5.03 ± 0.107^Cd^	5.59 ± 0.060^Cc^	6.05 ± 0.040^Cb^	6.74 ± 0.123^Ca^
Lactic acid bacteria (log10 CFU/g)	Control	2.20 ± 0.095^Af^	3.54 ± 0.085^Ae^	4.53 ± 0.111^Ad^	5.22 ± 0.120^Ac^	5.64 ± 0.100^Ab^	7.71 ± 0.083^Aa^
	1%	2.21 ± 0.088^Bf^	2.35 ± 0.055^Be^	3.49 ± 0.036^Bd^	3.74 ± 0.075^Bc^	4.20 ± 0.070^Ab^	4.62 ± 0.080^Ba^
	2%	2.18 ± 0.036^Ce^	2.12 ± 0.020^Ce^	2.20 ± 0.040^Cd^	2.40 ± 0.132^Cc^	2.90 ± 0.188^Bb^	3.25 ± 0.060^Ca^
Enterobacteriaceae (log10 CFU/g)	Control	3.21 ± 0.050^Af^	3.87 ± 0.075^Ae^	4.11 ± 0.020^Ad^	4.74 ± 0.070^Ac^	5.45 ± 0.072^Ab^	6.17 ± 0.045^Aa^
	1%	3.28 ± 0.055^Ad^	3.55 ± 0.185^Bd^	3.91 ± 0.020^Ac^	4.34 ± 0.045^Bb^	4.85 ± 0.216^Ba^	5.12 ± 0.104^Ba^
	2%	3.22 ± 0.075^Ad^	3.42 ± 0.096^Bc^	3.70 ± 0.060^Ac^	4.13 ± 0.125^Bb^	4.51 ± 0.081^Ba^	4.84 ± 0.088^Ca^
H2S‐producing bacteria (log10 CFU/g)	Control	2.21 ± 0.050^Af^	3.45 ± 0.075^Ae^	4.55 ± 0.020^Ad^	5.33 ± 0.070^Ac^	6.71 ± 0.072^Ab^	7.44 ± 0.045^Aa^
	1%	2.33 ± 0.055^Af^	2.87 ± 0.185^Be^	3.45 ± 0.020^Bd^	4.11 ± 0.045^Bc^	5.25 ± 0.216^Bb^	5.85 ± 0.104^Ba^
	2%	2.25 ± 0.075^Ae^	2.61 ± 0.096^Bd^	3.12 ± 0.060^Cc^	3.56 ± 0.125^Cb^	4.26 ± 0.081^Ca^	4.06 ± 0.088^Ca^

*Note:* Results have been expressed as log_10_ CFU/g of muscle. Data are represented as Mean ± Standard deviation (*n* = 3). Different letters indicate significant differences between the treatment groups (*p* ≤ 0.05), with capital letters representing significant differences in treatments and small letters representing storage conditions..

#### 3.6.1 Total Viable Count (TVC)

Various studies suggest that a bacterial count ranging from 2 to 6 log_10_ CFU/g is considered acceptable for freshwater fish (Austin 2006; Volpe et al. 2015). The TVC for the control sample was 3.2 log_10_ CFU/g on day 0 and increased to approximately 8.6 log_10_ CFU/g by day 12 of refrigerated storage. However, the initial TVC for samples treated with 1% and 2% pine nut extract was lower, reaching around 7.4 and 6.5 log_10_ CFU/g, respectively. According to ICMSF (1986), the acceptable upper limit for TVC in fresh fish is 7 log_10_ CFU/g. It was reported that the concentration of 2% pine nut kernel extract significantly (*p* ≤ 0.05) reduced the TVC in the treated samples, extended the shelf life, and preserved the quality of rainbow trout fillets at the end of day 12. The notable reduction in TVC could be attributed to the antimicrobial effect of the polyphenolic compounds present in the extract on aerobic spoilage bacteria.

#### 3.6.2 Psychrotrophic Bacteria Count (PBC)

The PBC, which is a primary measure of total aerobic plate counts and monitors the quality of fish, was conducted. Psychrotrophs are Gram‐negative bacteria, with *Pseudomonas* species being the dominant microflora. These bacteria can cause food poisoning and spoilage, resulting in unpleasant odors and changes during cold storage, rendering the fish unsuitable for consumption. The results indicated that psychrotrophic bacteria generally followed the same trend as TVC in rainbow trout fillets. Initially, the PBC counts were 3.3 log_10_ CFU/g, and their growth rate in all samples showed an increasing pattern over time. The PBC counts in the control sample significantly increased compared to the treated samples (*p* ≤ 0.05). The findings showed an increase in the initial PBC count (day 0) observed until day 20 of storage. However, the changes in the initial PBC for samples containing 1% and 2% pine nut extract were significantly lower (*p* ≤ 0.05) and showed a reduction of 6.8 and 6.05 log_10_ CFU/g on day 16, respectively. These results are consistent with those reported by Yu et al. (2017) and Socaciu et al. (2021), demonstrating that pine nut extract maintained the quality of rainbow trout fillets during refrigerated storage.

#### 3.6.3 Lactic Acid Bacteria (LAB)

The study examined the growth of LAB in both anaerobic and aerobic conditions. These bacteria are natural microbiota in fish and act as bio‐preservatives, inhibiting pathogenic and food‐borne microorganisms in fresh rainbow trout. The changes in the microbial state of fish fillets depend on the treatment and storage period. The initial LAB count for the control sample was 2.20 log_10_ CFU/g (day 0) and increased to 6.0 log_10_ CFU/g on day 20 of storage at 4°C. In contrast, the initial LAB count for samples containing 1% and 2% pine nut extract was significantly lower (*p* ≤ 0.05), showing reductions of 4.62 and 3.25 log_10_ CFU/g on day 20, respectively. The outcomes aligned with prior research by Nobahar et al. (2022) and Volpe et al. (2015). The data also revealed that while the initial LAB count increased over time for the control and samples with 1% and 2% pine nut extract, the increase was significantly lower for the treated samples (*p* ≤ 0.05). This decrease in LAB count is attributed to the impact of cold storage on the growth rate and the competition for nutrients between LAB and Pseudomonas in aerobic conditions (Nilsson and Gram 2002). LAB, characterized as Gram‐positive bacteria, produce lactic acid through fermentative processes and are a natural component of the microflora in healthy fish (Banerjee and Ray 2017). Numerous studies have indicated that LAB can generate certain off‐odors in fish products and lower their pH levels due to lactic acid production (Li et al. 2019; Chen et al. 2016). These bacteria belong to benign bacteriocin‐producing strains and may inhibit the growth of harmful microorganisms, thereby reducing spoilage by exerting inhibitory effects on spoilage‐related microbiota (Chaillou et al. 2014). Additionally, research has demonstrated that pine nut extract's antimicrobial activity can diminish LAB's growth, potentially leading to a decrease in off‐odor production. This reduction in undesirable odors may enhance the sensory acceptance of fish products by improving their aroma profile.

#### 3.6.4 Enterobacteriaceae

Enterobacteriaceae are considered a hygiene indicator and part of the microflora found in fresh rainbow trout. Similar to psychrotrophic bacteria, Enterobacteriaceae can thrive at low temperatures. However, their population does not show a significant increase during the refrigerated storage of food samples, as they exhibit a slower growth rate compared to other Gram‐negative psychrotrophic bacteria responsible for spoilage. These findings are consistent with studies by Özogul, Özogul, and Gökbulut. (2006) and Sathivel et al. (2007). The initial Enterobacteriaceae count for the control sample was 3.21 log_10_ CFU/g on day 0 and reached 6.17 log_10_ CFU/g after 20 days of storage. However, the initial Enterobacteriaceae count for samples treated with 1% and 2% pine nut extract remained lower than the control sample, showing a reduction of 5.12 and 4.84 log_10_ CFU/g, respectively, on day 20 of storage. The observed reduction in the microbial population in this study aligns with the results reported by Chytiri et al. (2004) and Mehdizadeh et al. (2019). Results revealed that the Enterobacteriaceae counts for the treated samples significantly decreased compared to the control (*p* ≤ 0.05). The results demonstrated that rainbow trout fillets treated with 2% pine nut extract significantly reduced the growth rate of Enterobacteriaceae (*p* ≤ 0.05). Our findings are in line with those obtained by Özogul, Kus, and Kuley (2013), who reported a reduction in Enterobacteriaceae count to below 7 log_10_ CFU/g in trout fillets treated with strawflower extract after 23 days of refrigerated storage.

#### 3.6.5 Hydrogen Sulfide (H_2_S)‐producing Bacteria

H_2_S‐producing bacteria contribute to fish spoilage by causing unpleasant off‐odors (Gram, Trolle, and Huss 1987; Nychas and Drosinos 2009). These bacteria, along with *Pseudomonas*, are recognized as competitive microorganisms. The initial delay in the growth of H_2_S‐producing bacteria may be due to the inhibitory effects of *Pseudomonas*. These bacteria play a significant role in spoilage as they increase TVB‐N levels and proteolytic enzymes, resulting in undesirable flavors (Ninan et al. 2011). The initial population of the H_2_S‐producing bacteria in the samples under study was approximately 2 log_10_ CFU/g (day 0). The findings showed an increase in the H_2_S‐producing bacteria throughout the following storage days. The count of H_2_S‐producing bacteria in the treated samples decreased significantly compared to the control sample from day 4 to day 20 of storage (*p* ≤ 0.05). In the sample containing 2% pine nut extract, the counts of H_2_S‐producing bacteria were approximately 2 log_10_ CFU/g, significantly (*p* ≤ 0.05) lower than the control sample with 5 log_10_ CFU/g on day 20 of storage. These results indicated that pine nut extract inhibited the growth of H_2_S‐producing bacteria in rainbow trout fillets during the storage period. These findings align with previous studies by Volpe et al. (2015) and Alizadeh Amoli et al. (2019). Furthermore, considering the acceptable limit of H_2_S reported in the literature to be 7 log_10_ CFU/g (Gram and Huss 1996), the 2% pine nut extract concentration maintained the quality of rainbow trout fillets within acceptable limits up to day 12 of storage.

### 3.7 Sensory Evaluation

The incorporation of pine nut extract, particularly at higher concentrations of 2%, into the rainbow trout fillets significantly improved (*p* ≤ 0.05) their color, odor, and taste, thereby extending their shelf life while preserving quality (Table 4). Fillets treated with pine nut extract exhibited reduced fishy odor and taste. Huang et al. (2018) reported similar results, noting improved sensory aroma and flavor characteristics in grass carp fillets after treatment with oregano essential oil. The color sensory scores notably decreased (*p* ≤ 0.05) for both control and treated samples during storage. A score higher than 3 was considered acceptable, and consequently, all acceptability standards for control samples were not met by day 8 of storage at 4°C ± 1°C. However, the acceptability limits were met after 8 and 12 days for samples treated with 1% and 2% pine nut extracts, respectively (Figure 1B). A study by Tokur et al. (2016) discovered that coating rainbow trout fillets with protein isolate enriched with thyme essential oils at higher concentrations led to extended shelf life and improved sensory attributes during refrigerated storage. The sensory scores for color showed significant differences between the control and treated samples throughout the storage period, with the treated samples exhibiting significantly higher scores, except for color on day 4. Additionally, samples treated with 1% and 2% pine nut extracts showed similar acceptability for color on days 4 and 8 of refrigerated storage, respectively. Consistent with these findings, other studies by Takyar, Haghighat Khajavi, and Safari (2019) and Sáez et al. (2021) highlighted that adding algae extracts extended the shelf life of rainbow trout fillets, attributed to the considerable antioxidant activities during refrigerated storage. These results indicated that the shelf‐life extension of rainbow trout fillets was due to the antioxidant effects of the phenolic compounds in pine nut extract, strengthening the findings of chemical quality analyses.

**TABLE 4:** Sensory properties of rainbow trout fillets stored at 4°C ± 1°C for 20 days.

Attributes	Treatments	Storage days					
		0	4	8	12	16	20
Color	Control	4.99 ± 0.02^Aa^	3.90 ± 0.30^Bb^	2.90 ± 0.25^Cb^	2.12 ± 0.27^Db^	1.46 ± 0.21^Eb^	1.12 ± 0.13^Eb^
	1%	4.98 ± 0.01^Aa^	4.24 ± 0.17^Ba^	3.54 ± 0.21^Ca^	2.82 ± 0.19^Da^	2.20 ± 0.10^Eb^	1.56 ± 0.18^Fb^
	2%	4.98 ± 0.02^Aa^	4.30 ± 0.19^Ba^	3.54 ± 0.21^Ca^	2.86 ± 0.23^Da^	2.18 ± 0.16^Ea^	1.64 ± 0.11^Fa^
Odor	Control	4.98 ± 0.01^Aa^	4.24 ± 0.17^Bb^	2.34 ± 0.23^Cb^	1.20 ± 0.16^Db^	1.01 ± 0.01^Ec^	1.00 ± 0.00^Ec^
	1%	4.97 ± 0.02^Aa^	4.20 ± 0.16^Bb^	3.66 ± 0.15^Ca^	2.84 ± 0.21^Da^	1.94 ± 0.42^Eb^	1.01 ± 0.01^Fb^
	2%	4.99 ± 0.01^Aa^	4.54 ± 0.11^Ba^	3.88 ± 0.22^Ca^	3.08 ± 0.29^Da^	2.30 ± 0.16^Ea^	1.56 ± 0.17^Fa^
Taste	Control	4.99 ± 0.01^Aa^	4.10 ± 0.12^Bb^	2.24 ± 0.11^Cc^	1.80 ± 0.08^Dc^	1.02 ± 0.00^Eb^	1.01 ± 0.00^Eb^
	1%	4.96 ± 0.01^Aa^	4.22 ± 0.13^Bb^	3.76 ± 0.13^Cb^	2.92 ± 0.18^Db^	2.08 ± 0.23^Ea^	1.10 ± 0.10^Fb^
	2%	4.98 ± 0.02^Aa^	4.52 ± 0.16^Ba^	4.06 ± 0.24^Ca^	3.36 ± 0.25^Da^	2.28 ± 0.15^Ea^	1.82 ± 0.08^Fa^

*Note:* Data are represented as Mean ± Standard deviation (*n* = 10). Different letters indicate significant differences between the treatment groups (*p* ≤ 0.05), with capital letters representing significant differences in treatments and small letters representing storage conditions.

## 4 Conclusions

The application of edible coatings shows significant promise for food preservation. The present study revealed that rainbow trout fillets immersed in pine nut defatted extract stored under refrigeration maintained acceptable levels of TVB‐N, TBA values, and free fatty acid content for up to 8 days with 1% and 12 days with 2% pine nut extract. The study also found that pine nut extract, particularly at higher concentrations, exhibited greater DPPH radical scavenging capacity, thereby preserving the freshness of trout fillets by reducing discoloration and deterioration due to oxidation during cold storage. Sensory evaluation confirmed that samples treated with 2% extract remained within acceptable limits for up to 12 days of refrigerated storage. These findings suggest that pine nut extract could be a promising natural antioxidant and bio‐preservative which can effectively delay oxidation and microbial spoilage, maintain sensory characteristics, and extend the shelf life of refrigerated rainbow trout fillets.

## Author Contributions

**Shaghayegh Soleimani:** formal analysis (equal), investigation (lead), resources (lead), software (lead), writing – original draft (equal). **Shabnam Haghighat Khajavi:** conceptualization (lead), data curation (lead), formal analysis (equal), investigation (supporting), methodology (equal), project administration (lead), software (supporting), supervision (lead), validation (lead), visualization (lead), writing – original draft (equal), writing – review and editing (lead). **Reza Safari:** methodology (equal), resources (supporting), supervision (supporting).

## Ethics Statement

The authors have nothing to report.

## Conflicts of Interest

The authors declare no conflicts of interest.

## Data Availability

Data are available on request from the author.

## References

[fsn34685-bib-0001] Alirezalu, K. , M. Yaghoubi , Z. Nemati , B. Farmani , and A. Mousavi Khaneghah . 2021. “Efficacy of Stinging Nettle Extract in Combination With ε‐Polylysine on the Quality, Safety, and Shelf Life of Rainbow Trout Fillets.” Food Science & Nutrition 9, no. 3: 1542–1550. 10.1002/fsn3.2129. 33747468 PMC7958555

[fsn34685-bib-0002] Alizadeh Amoli, Z. , T. Mehdizadeh , H. Tajik , and M. Azizkhani . 2019. “Shelf‐Life Extension of Refrigerated, Vacuum‐Packed Rainbow Trout Dipped in an Alginate Coating Containing an Ethanolic Extract and/or the Essential Oil Mentha Aquatic.” Chemical Papers 73, no. 10: 2541–2550. 10.1007/s11696-019-00808-8.

[fsn34685-bib-0003] Amorati, R. , M. C. Foti , and L. Valgimigli . 2013. “Antioxidant Activity of Essential Oils.” Journal of Agriculture and Food Chemistry 61, no. 46: 10835–10847. 10.1021/jf403496k. 24156356

[fsn34685-bib-0004] AOCS . 2017a. “Official Method (7th Ed.) cd 8b‐90, Peroxide Value, Acetic Acid, Isooctane Method.” In Official Methods and Recommended Practices of the American Oil Chemists' Society. Champaign, IL, USA: AOCS press.

[fsn34685-bib-0005] AOCS . 2017b. “Official Method (7th Ed.) cd 19‐90, 2‐Thiobarbituric Acid Value, Direct Method.” In Official Methods and Recommended Practices of the American Oil Chemists' Society. Champaign, IL, USA: AOCS press.

[fsn34685-bib-0006] AOCS . 2017c. “Official Method (7th Ed.) cd 3d‐63, Acid Value of Fats and Oils.” In Official Methods and Recommended Practices of the American Oil Chemists' Society. Champaign, IL, USA: AOCS press.

[fsn34685-bib-0007] Arashisar, Ş. , O. Hisar , M. Kaya , and T. Yanik . 2004. “Effects of Modified Atmosphere and Vacuum Packaging on Microbiological and Chemical Properties of Rainbow Trout (Oncorynchus Mykiss) Fillets.” International Journal of Food Microbiology 97, no. 2: 209–214. 10.1016/j.ijfoodmicro.2004.05.024. 15541807

[fsn34685-bib-0008] Austin, B. 2006. “The Bacterial Microflora of Fish: Review Article.” Scientific World Journal 6, no. 11: 931–945. 10.1100/tsw.2006.181. 16906326 PMC5917212

[fsn34685-bib-0009] Banerjee, G. , and A. K. Ray . 2017. “Bacterial Symbiosis in the Fish Gut and Its Role in Health and Metabolism.” Symbiosis 72, no. 1: 1–11. 10.1007/s13199-016-0441-8.

[fsn34685-bib-0010] Barthet, V. J. , V. Gordon , and J. K. Daun . 2008. “Evaluation of a Colorimetric Method for Measuring the Content of FFA in Marine and Vegetable Oils.” Food Chemistry 111, no. 4: 1064–1068. 10.1016/j.foodchem.2008.05.026.

[fsn34685-bib-0011] Bligh, E. G. , and W. J. Dyer . 1959. “A Rapid Method of Total Lipid Extraction and Purification.” Canadian Journal of Biochemistry and Physiology 37, no. 8: 911–917. 10.1139/o59-099. 13671378

[fsn34685-bib-0012] Bodoira, R. , and D. Maestri . 2020. “Phenolic Compounds From Nuts: Extraction, Chemical Profiles, and Bioactivity.” Journal of Agricultural and Food Chemistry 68, no. 4: 927–942. 10.1021/acs.jafc.9b07160. 31910006

[fsn34685-bib-0013] Boselli, E. , M. F. Carboni , M. T. Rodriguez‐Estrada , T. G. Toschi , M. Daniel , and G. Lercker . 2005. “Photoxidation of Cholesterol and Lipids of Turkey Meat During Storage Under Commercial Retail Conditions.” Food Chemistry 91: 705–713. 10.1016/j.foodchem.2004.06.043.

[fsn34685-bib-0014] Botsoglou, N. A. , D. J. Fletouris , G. E. Papageorgiou , V. N. Vassilopoulos , A. J. Mantis , and A. G. Trakatellis . 1994. “Rapid, Sensitive, and Specific Thiobarbituric Acid Method for Measuring Lipid Peroxidation in Animal Tissue, Food, and Feedstuff Samples.” Journal of Agricultural and Food Chemistry 42, no. 9: 1931–1937. 10.1021/jf00045a019.

[fsn34685-bib-0015] Brand‐Williams, W. , M. E. Cuvelier , and C. L. W. T. Berset . 1995. “Use of a Free Radical Method to Evaluate Antioxidant Activity.” LWT‐ Food Science and Technology 28, no. 1: 25–30. 10.1016/S0023-6438(95)80008-5.

[fsn34685-bib-0016] Chaillou, S. , S. Christieans , M. Rivollier , I. Lucquin , M. C. Champomier‐Verges , and M. Zagorec . 2014. “Quantification and Efficiency of *Lactobacillus sakei* Strain Mixtures Used as Protective Cultures in Ground Beef.” Meat Science 97, no. 3: 332–338. 10.1016/j.meatsci.2013.08.009. 24041591

[fsn34685-bib-0017] Chen, B. J. , Y. J. Zhou , X. Y. Wei , H. J. Xie , R. C. Hider , and T. Zhou . 2016. “Edible Antimicrobial Coating Incorporating a Polymeric Iron Chelator and Its Application in the Preservation of Surimi Product.” Food and Bioprocess Technology 9, no. 6: 1031–1039. 10.1007/s11947-016-1693-2.

[fsn34685-bib-0018] Chytiri, S. , I. Chouliara , I. N. Savvaidis , and M. G. Kontominas . 2004. “Microbiological, Chemical and Sensory Assessment of Iced Whole and Filleted Aquacultured Rainbow Trout.” Food Microbiology 21, no. 2: 157–165. 10.1016/S0740-0020(03)00059-5.

[fsn34685-bib-0019] Erkan, N. 2012. “The Effect of Thyme and Garlic Oil on the Preservation of Vacuum‐Packaged Hot Smoked Rainbow Trout ( *Oncorhynchus mykiss* ).” Food and Bioprocess Technology 5: 1246–1254. 10.1007/s11947-010-0412-7.

[fsn34685-bib-0020] European Commission . 2005. “Commission Regulation (EC) No 2074/2005. Determination of the Concentration of TVB‐N in Fish and Fishery Products.” Official Journal of the European Union L338/37: 37–39.

[fsn34685-bib-0021] European Commission . 2008. “Commission Regulation (EC) No 1022/2008. Amending Regulation (EC) no 2074/2005 as Regards the Total Volatile Basic Nitrogen (TVB‐N) Limits.” Official Journal of the European Union L277/20: 18–20.

[fsn34685-bib-0022] European Commission . 2017. “Commission Implementing Regulation (EU) 2017/1925.” Official Journal of the European Union L282/334: 97.

[fsn34685-bib-0023] Fan, W. , Y. Chi , and S. Zhang . 2008. “The Use of a Tea Polyphenol Dip to Extend the Shelf Life of Silver Carp (Hypophthalmicthys Molitrix) During Storage in Ice.” Food Chemistry 108, no. 1: 148–153. 10.1016/j.foodchem.2007.10.057.

[fsn34685-bib-0024] Ghaly, A. E. , D. Dave , S. Budge , and M. S. Brooks . 2010. “Fish Spoilage Mechanisms and Preservation Techniques: Review.” American Journal of Applied Sciences 7, no. 7: 859–877. 10.3844/ajassp.2010.859.877.

[fsn34685-bib-0025] Giménez, B. , P. Roncalés , and J. A. Beltrán . 2002. “Modified Atmosphere Packaging of Filleted Rainbow Trout.” Journal of the Science of Food and Agriculture 82, no. 10: 1154–1159. 10.1002/jsfa.1136.

[fsn34685-bib-0026] Goulas, A. E. , and M. G. Kontominas . 2007a. “Combined Effect of Light Salting, Modified Atmosphere Packaging and Oregano Essential Oil on the Shelf‐Life of Sea Bream ( *Sparus aurata* ): Biochemical and Sensory Attributes.” Food Chemistry 100, no. 1: 287–296. 10.1016/j.foodchem.2005.09.045.

[fsn34685-bib-0027] Goulas, A. E. , and M. G. Kontominas . 2007b. “Effect of Modified Atmosphere Packaging and Vacuum Packaging on the Shelf‐Life of Refrigerated Chub Mackerel ( *Scomber japonicus* ): Biochemical and Sensory Attributes.” European Food Research and Technology 224, no. 5: 545–553. 10.1007/s00217-006-0316-y.

[fsn34685-bib-0028] Gram, L. , and H. H. Huss . 1996. “Microbiological Spoilage of Fish and Fish Products.” International Journal of Food Microbiology 33, no. 1: 121–137. 10.1016/0168-1605(96)01134-8. 8913813

[fsn34685-bib-0029] Gram, L. , G. Trolle , and H. H. Huss . 1987. “Detection of Specific Spoilage Bacteria From Fish Stored at Low (0°C) and High (20°C) Temperatures.” International Journal of Food Microbiology 4, no. 1: 65–72. 10.1016/0168-1605(87)90060-2.

[fsn34685-bib-0030] Hoon, L. Y. , C. Choo , M. I. Watawana , N. Jayawardena , and V. Y. Waisundara . 2015. “Evaluation of the Total Antioxidant Capacity and Antioxidant Compounds of Different Solvent Extracts of Chilgoza Pine Nuts ( *Pinus gerardiana* ).” Journal of Functional Foods 18: 1014–1021. 10.1016/j.jff.2014.07.009.

[fsn34685-bib-0031] Huang, Z. , X. Liu , S. Jia , L. Zhang , and Y. Luo . 2018. “The Effect of Essential Oils on Microbial Composition and Quality of Grass Carp (*Ctenopharyngodon idellus*) Fillets During Chilled Storage.” International Journal of Food Microbiology 266: 52–59. 10.1016/j.ijfoodmicro.2017.11.003. 29175764

[fsn34685-bib-0032] ICMSF (International Commission on Microbiological Specifications of Foods) . 1986. “Sampling Plans for Fish and Shellfish.” In Microorganisms in Foods. Sampling for Microbiological Analysis: Principles and Specific Applications, vol. 2, 2nd ed., 181–196. Toronto, ON, Canada: University of Toronto Press.

[fsn34685-bib-0033] ISO 21528‐2 . 2004. “Microbiology of Food and Animal Feeding Stuffs‐Horizontal Methods for the Detection and Enumeration of Enterobacteriaceae—Part 2: Colony Count Technique.”

[fsn34685-bib-0034] ISO 4833‐1:2013/Amd 1 . 2022. “Microbiology of the Food Chain‐Horizontal Method for the Enumeration of Microorganisms‐Part 1: Colony Count at 30°C by the Pour Plate Technique. ISO: Geneva, Switzerland.”

[fsn34685-bib-0035] Jasour, M. S. , A. Ehsani , L. Mehryar , and S. S. Naghibi . 2015. “Chitosan Coating Incorporated With the Lactoperoxidase System: An Active Edible Coating for Fish Preservation.” Journal of the Science of Food and Agriculture 95, no. 6: 1373–1378. 10.1002/jsfa.6838. 25060563

[fsn34685-bib-0036] Kołakowska, A. , Z. Domiszewski , D. Kozłowski , and M. Gajowniczek . 2006. “Effects of Rainbow Trout Freshness on n‐3 Polyunsaturated Fatty Acids in Fish Offal.” European Journal of Lipid Science and Technology 108, no. 9: 723–729. 10.1002/ejlt.200600054.

[fsn34685-bib-0037] Kornsteiner, M. , K. H. Wagner , and I. Elmadfa . 2006. “Tocopherols and Total Phenolic in 10 Different Nut Types.” Food Chemistry 98, no. 2: 381–387. 10.1016/j.foodchem.2005.07.033.

[fsn34685-bib-0038] Li, J. , X. Yang , G. Shi , J. Chang , Z. Liu , and M. Zeng . 2019. “Cooperation of Lactic Acid Bacteria Regulated by the AI‐2/LuxS System Involve in the Biopreservation of Refrigerated Shrimp.” Food Research International 120: 679–687. 10.1016/j.foodres.2018.11.025. 31000286

[fsn34685-bib-0039] Lim, J. 2011. “Hedonic Scaling: A Review of Methods and Theory.” Food Quality and Preference 22, no. 8: 733–747. 10.1016/j.foodqual.2011.05.008.

[fsn34685-bib-0040] Mehdizadeh, T. , H. Tajik , S. Jafarie , and A. Kaboudari . 2019. “Effect of *Salvia officinalis* L. Extract on Chemical, Microbial, Sensory and Shelf Life of Rainbow Trout Fillet.” Food Science and Biotechnology 28, no. 5: 1499–1506. 10.1007/s10068-019-00575-y. 31695949 PMC6811673

[fsn34685-bib-0041] Mi, H. , X. Guo , and J. Li . 2016. “Effect of 6‐Gingerol as Natural Antioxidant on the Lipid Oxidation in Red Drum Filles During Refrigerated Storage.” LWT‐ Food Science and Technology 74: 70–76. 10.1016/j.lwt.2016.07.029.

[fsn34685-bib-0042] Miraliakbari, H. , and F. Shahidi . 2008. “Oxidative Stability of Free Nut Oils.” Journal of Agriculture and Food Chemistry 56, no. 22: 4751–4759. 10.1021/jf8000982. 18494484

[fsn34685-bib-0043] Mohan, C. O. , C. N. Ravishankar , and T. K. Srinivasagopal . 2008. “Effect of O2 Scavenger on the Shelf‐Life of Catfish ( *Pangasius sutchi* ) Steaks During Chilled Storage.” Journal of the Science of Food and Agriculture 88, no. 3: 442–448. 10.1002/jsfa.3105.

[fsn34685-bib-0044] Nie, X. , R. Zhang , L. Cheng , W. Zhu , S. Li , and X. Chen . 2022. “Mechanisms Underlying the Deterioration of Fish Quality After Harvest and Methods of Preservation.” Food Control 135: 108805. 10.1016/j.foodcont.2021.108805.

[fsn34685-bib-0045] Nilsson, L. , and L. Gram . 2002. “Improving the Control of Pathogens in Fish Products.” In Safety and Quality Issues in Fish Processing, edited by H. A. Bremner , 54–84. Cambridge, England: Woodhead Publishing in Food Science and Technology.

[fsn34685-bib-0046] Ninan, G. , K. V. Lalitha , A. A. Zynudheen , and J. Joseph . 2011. “Effect of Chilling on Microbiological, Biochemical and Sensory Attributes of Whole Aquaculture Rainbow Trout ( *Oncorhynchus mykiss* Walbaum, 1792).” Journal of Aquatic Research and Development 6: 1–8. 10.4172/2155-9546.S5-001.

[fsn34685-bib-0047] Nobahar, S. , S. Haghighat Khajavi , and R. Safari . 2022. “Effect of Whey Protein Concentrate Coating Enriched With Spearmint Essential Oil on Oxidation and Microbial Spoilage of Minced Common Kilka During Refrigerated Storage.” Science, Engineering and Health Studies 116: 1–10. 10.14456/sehs.2022.1.

[fsn34685-bib-0048] Nychas, G. J. E. , and E. H. Drosinos . 2009. “Detection of Fish Spoilage.” In Handbook of Seafood and Seafood Products Analysis, edited by M. L. Nollet Leo and F. Toldra , 537–555. Boca Raton, FL, USA: CRC Press: Taylor & Francis Group. 10.1201/9781420046359.

[fsn34685-bib-0049] Ojagh, S. M. , M. Rezaei , S. H. Razavi , and S. M. H. Hosseini . 2010. “Effect of Chitosan Coatings Enriched With Cinnamon Oil on the Quality of Refrigerated Rainbow Trout.” Food Chemistry 120, no. 1: 193–198. 10.1016/j.foodchem.2009.10.006.

[fsn34685-bib-0050] Ólafsdóttir, G. , and R. Jónsdóttir . 2009. “Volatile Aroma Compounds in Fish.” In Handbook of Seafood and Seafood Products Analysis, edited by L. M. L. Nollet and F. Toldra , 97–117. Boca Raton, FL, USA: CRC Press: Taylor & Francis Group. 10.1201/9781420046359.

[fsn34685-bib-0051] Oraei, M. , A. Motalebi , E. Hoseini , and S. Javan . 2011. “Effect of Gamma Irradiation and Frozen Storage on Microbial Quality of Rainbow Trout ( *Oncorhynchus mykiss* ) Fillet.” Iranian Journal of Fisheries Sciences 10, no. 1: 75–84. 10.1111/j.1365-2621.2011.02930.x.

[fsn34685-bib-0052] Oroian, M. , and I. Escriche . 2015. “Antioxidants: Characterization, Natural Sources, Extraction and Analysis.” Food Research International 74: 10–36. 10.1016/j.foodres.2015.04.018. 28411973

[fsn34685-bib-0053] O'Sullivan, A. , A. Mayr , N. Shaw , S. Murphy , and J. Kerry . 2005. “Use of Natural Antioxidants to Stabilize Fish Oil Systems.” Journal of Aquatic Food Product Technology 14, no. 3: 75–94. 10.1300/J030v14n03_06.

[fsn34685-bib-0054] Özogul, F. , B. Kus , and E. Kuley . 2013. “The Impact of Strawflower and Mistletoe Extract on Quality Properties of Rainbow Trout Fillets.” International Journal of Food Science and Technology 48, no. 1: 2228–2238. 10.1111/ijfs.12209.

[fsn34685-bib-0055] Özogul, Y. , F. Özogul , and C. Gökbulut . 2006. “Quality Assessment of Wild European Eel ( *Anguilla Anguilla* ) Stored in Ice.” Food Chemistry 95, no. 3: 458–465. 10.1016/j.foodchem.2005.01.025.

[fsn34685-bib-0056] Pearson, A. , J. Gray , A. M. Wolzak , and N. Horenstein . 1983. “Safety Implications of Oxidized Lipids in Muscle Foods.” Food Technology 37, no. 7: 121–129.

[fsn34685-bib-0057] Pezeshk, S. , M. Rezaei , and H. Hosseini . 2011. “Effect of Turmeric, Shallot Extracts, and Their Combination on Quality Characteristics of Vacuum‐Packaged Rainbow Trout Stored at 4°C±1°C.” Journal of Food Science 76, no. 6: 387–391. 10.1111/j.1750-3841.2011.02242.x. 21729071

[fsn34685-bib-0058] Pirozzi, A. , G. Pataro , F. Donsì , and G. Ferrari . 2021. “Edible Coating and Pulsed Light to Increase the Shelf Life of Food Products.” Food Engineering Reviews 13: 544–569. 10.1007/s12393-020-09245-w.

[fsn34685-bib-0059] Quitral, V. , M. L. Donoso , J. Ortiz , M. V. Herrera , H. Araya , and S. P. Aubourg . 2009. “Chemical Changes During the Chilled Storage of Chilean Jack Mackerel ( *Trachurus murphyi* ): Effect of a Plant‐Extract Icing System.” LWT‐ Food Science and Technology 42, no. 8: 1450–1454. 10.1016/j.lwt.2009.03.005.

[fsn34685-bib-0060] Rusted, T. 2009. “Lipid Oxidation.” In Handbook of Seafood and Seafood Products Analysis, edited by M. L. Nollet Leo and F. Toldra , 87–95. Boca Raton, FL, USA: CRC Press: Taylor & Francis Group. 10.1201/9781420046359.

[fsn34685-bib-0061] Sáez, M. I. , M. D. Suárez , F. J. Alarón , and T. F. Martínez . 2021. “Assessing the Potential of Algae Extracts for Extending the Shelf Life of Rainbow Trout ( *Oncorhynchus mykiss* ) Fillets.” Food 10, no. 5: 910–922. 10.3390/foods10050910. PMC8143106 33919226

[fsn34685-bib-0062] Sallam, K. I. , A. Ahmed , M. Elgazzar , and E. Eldaly . 2007. “Chemical Quality and Sensory Attributes of Marinated Pacific Saury ( *Cololabis saira* ) During Vacuum‐Packaged Storage at 4°C.” Food Chemistry 102, no. 4: 1061–1070. 10.1016/j.foodchem.2006.06.044.

[fsn34685-bib-0063] Sathe, S. K. , E. K. Monaghan , H. H. Kshirsagar , and M. Venkatachalam . 2008. “Chemical Composition of Edible Nut Seeds and Its Implications in Human Health.” In Tree Nuts: Composition, Phytochemicals and Health Effects, edited by C. Alasalvar and F. Shahidi , 12–35. Boca Raton, FL, USA: CRC Press: Taylor & Francis Group. 10.1201/9781420019391.

[fsn34685-bib-0064] Sathivel, S. , Q. Liu , J. Huang , and W. Prinyawiwatkul . 2007. “The Influence of Chitosan Glazing on the Quality of Skinless Pink Salmon ( *Oncorhynchus gorbuscha* ) Fillets During Frozen Storage.” Journal of Food Engineering 83, no. 3: 366–373. 10.1016/j.jfoodeng.2007.03.009.

[fsn34685-bib-0065] Secci, G. , and G. Parisi . 2016. “From Farm to Fork: Lipid Oxidation in Fish Products. A Review.” Italian Journal of Animal Science 15, no. 1: 124–136. 10.1080/1828051X.2015.1128687.

[fsn34685-bib-0066] Senturk Parreidt, T. , K. Müller , and M. Schmid . 2018. “Alginate‐Based Edible Films and Coatings for Food Packaging Applications.” Food 7, no. 10: 170. 10.3390/foods7100170. PMC6211027 30336642

[fsn34685-bib-0067] Shahidi, F. , and C. Alsalvar . 2008. “Tree Nuts: Composition, Phytochemicals, and Health Effects: An Overview.” In Tree Nuts: Composition, Phytochemicals and Health Effects, edited by C. Alasalvar and F. Shahidi . Boca Raton, FL, USA: CRC Press: Taylor & Francis Group. 10.1201/9781420019391.

[fsn34685-bib-0068] Shahidi, F. , and P. Ambigaipalan . 2015. “Phenolics and Polyphenolics in Foods, Beverages and Spices: Antioxidant Activity and Health Effects‐ A Review.” Journal of Functional Foods 18: 820–897. 10.1016/j.jff.2015.06.018.

[fsn34685-bib-0069] Sharma, A. , R. Goyal , and L. Sharma . 2016. “Potential Biological Efficacy of Pinus Plant Species Against Oxidative, Inflammatory and Microbial Disorders.” BMC Complementary and Alternative Medicine 16: 35. 10.1186/s12906-016-1011-6. 26822870 PMC4730770

[fsn34685-bib-0070] Shewfelt, R. L. 1981. “Fish Muscle Lipolysis‐A Review.” Journal of Food Biochemistry 5, no. 2: 79–100. 10.1111/j.1745-4514.1981.tb00663.x.

[fsn34685-bib-0071] Singh, G. , D. Kumar , and A. K. Dash . 2021. “ *Pinus gerardiana* Wallichex.” D. Don. ‐A Review. Phytomedicine Plus 1, no. 2: 100024. 10.1016/j.phyplu.2021.100024.

[fsn34685-bib-0072] Socaciu, M. I. , M. Fogarasi , E. L. Simon , et al. 2021. “Effects of Whey Protein Isolate‐Based Film Incorporated With Tarragon Essential Oil on the Quality and Shelf‐Life of Refrigerated Brook Trout.” Food 10, no. 2: 401–424. 10.3390/foods10020401. PMC7918404 33670385

[fsn34685-bib-0073] Song, H. Y. , Y. J. Shin , and K. B. Song . 2012. “Preparation of a Barley Bran Protein‐Gelatin Composite Film Containing Grapefruit Seed Extract and Its Application in Salmon Packaging.” Journal of Food Engineering 113: 541–547. 10.1016/j.jfoodeng.2012.07.010.

[fsn34685-bib-0074] Suhag, R. , N. Kumar , A. T. Petkoska , and A. Upadhyay . 2020. “Film Formation and Deposition Methods of Edible Coating on Food Products: A Review.” Food Research International 136: 109582. 10.1016/j.foodres.2020.109582. 32846613

[fsn34685-bib-0075] Tajkarimi, M. , and S. A. Ibrahim . 2012. “Phytochemicals as Anti‐Microbial Food Preservatives.” In Dietary Phytochemicals and Microbes, edited by A. K. Patra , 207–235. Dordrecht, Netherland: Springer. 10.1007/978-94-007-3926-0.

[fsn34685-bib-0076] Takyar, M. B. T. , S. Haghighat Khajavi , and R. Safari . 2019. “Evaluation of Antioxidant Properties of Chlorella Vulgaris and Spirulina Platensis and Their Application in Order to Extend the Shelf Life of Rainbow Trout ( *Oncorhynchus mykiss* ) Fillets During Refrigerated Storage.” LWT‐ Food Science and Technology 100: 244–249. 10.1016/j.lwt.2018.10.079.

[fsn34685-bib-0077] Thilakarathna, R. C. N. , L. F. Siow , T. K. Tang , and Y. Y. Lee . 2023. “A Review on Application of Ultrasound and Ultrasound Assisted Technology for Seed Oil Extraction.” Journal of Food Science and Technology 60, no. 4: 1222–1236. 10.1007/s13197-022-05359-7. 36936117 PMC10020383

[fsn34685-bib-0078] Tokur, B. K. , F. Sert , E. T. Aksun , and F. Özoğul . 2016. “The Effect of Whey Protein Isolate Coating Enriched With Thyme Essential Oils on Trout Quality at Refrigerated Storage (4±2°C).” Journal of Aquatic Food Product Technology 25, no. 4: 585–596. 10.1080/10498850.2014.896063.

[fsn34685-bib-0079] Trbović, D. , D. Vranić , J. Đinović‐Stojanović , et al. 2012. “Fatty Acid Profile in Rainbow Trout ( *Oncorhynchus mykiss* ) as Influenced by Diet.” Biotechnology in Animal Husbandry 28, no. 3: 563–573. 10.2298/BAH1203563T.

[fsn34685-bib-0080] Uçak, I. , R. Khalily , C. Carrillo , I. Tomasevic , and F. J. Barba . 2020. “Potential of Propolis Extract as a Natural Antioxidant and Antimicrobial in Gelatin Films Applied to Rainbow Trout ( *Oncorhynchus mykiss* ) Fillets.” Food 9, no. 11: 1584. 10.3390/foods9111584. PMC7693740 33139596

[fsn34685-bib-0081] Uçak, İ. , Y. Özogul , and M. Durmuş . 2011. “The Effects of Rosemary Extract Combination With Vacuum Packing on the Quality Changes of Atlantic Mackerel Fish Burgers.” International Journal of Food Science and Technology 46, no. 6: 1157–1163. 10.1111/j.1365-2621.2011.02610.x.

[fsn34685-bib-0082] Volpe, M. G. , F. Siano , M. Paolucci , et al. 2015. “Active Edible Coating Effectiveness in Shelf‐Life Enhancement of Trout (*Oncorhynchusmykiss*) Fillets.” LWT‐ Food Science and Technology 60, no. 1: 615–622. 10.1016/j.lwt.2014.08.048.

[fsn34685-bib-0083] Wang, D. , H. Xiao , X. Lyu , H. Chen , and F. Wei . 2023. “Lipid Oxidation in Food Science and Nutritional Health: A Comprehensive Review.” Oil Crop Science 8, no. 1: 35–44. 10.1016/j.ocsci.2023.02.002.

[fsn34685-bib-0084] Wu, H. , S. Xiao , J. Yin , J. Zhang , and M. P. Richards . 2021. “Mechanisms Involved in the Inhibitory Effects of Free Fatty Acids on Lipid Peroxidation in Turkey Muscle.” Food Chemistry 342: 128333. 10.1016/j.foodchem.2020.128333. 33067046

[fsn34685-bib-0085] Yu, D. , Q. Jiang , Y. Xu , and W. Xia . 2017. “The Shelf‐Life Extension of Refrigerated Grass Carp (*Ctenopharyngodon idellus*) Fillets by Chitosan Coating Combined With Glycerol Monolaurate.” International Journal of Biological Macromolecules 101: 448–454. 10.1016/j.ijbiomac.2017.03.038. 28283457

[fsn34685-bib-0086] Yu, L. , and M. Slavin . 2008. “Nutraceutical Potential of Pine Nut.” In Tree Nuts: Composition, Phytochemicals and Health Effect, edited by C. Alasalvar and F. Shahidi , 285–295. Boca Raton, FL, USA: CRC Press: Taylor & Francis Group. 10.1201/9781420019391.

[fsn34685-bib-0087] Zhang, W. , J. Cao , X. Fan , and W. Jiang . 2020. “Applications of Nitric Oxide and Melatonin in Improving Postharvest Fruit Quality and the Separate and Crosstalk Biochemical Mechanisms.” Trends in Food Science and Technology 99: 531–541. 10.1016/j.tifs.2020.03.024.

[fsn34685-bib-0088] Zhou, Y. , Y. Zhang , J. Liang , et al. 2024. “From Formation to Solutions: Off‐Flavors and Innovative Removal Strategies for Farmed Freshwater Fish.” Trends in Food Science and Technology 144: 104318. 10.1016/j.tifs.2023.104318.

